# Human lung fibroblasts may modulate dendritic cell phenotype and function: results from a pilot *in vitro* study

**DOI:** 10.1186/s12931-016-0345-4

**Published:** 2016-04-04

**Authors:** Olivia Freynet, Joëlle Marchal-Sommé, Francette Jean-Louis, Arnaud Mailleux, Bruno Crestani, Paul Soler, Laurence Michel

**Affiliations:** Inserm U 1152, 46, rue Henri Huchard, Paris, 75018 France; Université Paris Diderot, Sorbonne Paris Cité, Paris, France; DHU FIRE, Paris, France; Assistance Publique-Hôpitaux de Paris, Hôpital Bichat, Service de Pneumologie A, Paris, France; Inserm UMR-S 976, Université Paris Diderot, Sorbonne Paris Cité, Hôpital Saint Louis, Paris, France; Service de Pneumologie, Hôpital Bichat, 46, rue Henri Huchard, Paris cedex 18, 75018 France; Inserm UMR-S 976, Hôpital Saint-Louis, 1 avenue Claude Vellefaux, 75475, Paris, 75010 France

**Keywords:** Lung, Fibroblasts, Dendritic cells, Fibrosis

## Abstract

In human lung fibrotic lesions, fibroblasts were shown to be closely associated with immature dendritic cell (DC) accumulation. The aim of the present pilot study was to characterize the role of pulmonary fibroblasts on DC phenotype and function, using co-culture of lung fibroblasts from patients with idiopathic pulmonary fibrosis (IPF) and from control patients, with a DC cell line MUTZ-3. We observed that co-culture of lung control and IPF fibroblasts with DCs reduced the expression of specific DC markers and down-regulated their T-cell stimulatory activity. This suggests that pulmonary fibroblasts might sustain chronic inflammation in the fibrotic lung by maintaining in situ a pool of immature DCs.

## Findings

### Background

We previously reported that in human fibrotic interstitial lung disease, immature DC accumulate in large numbers in areas of established fibrosis, in the close vicinity of fibroblasts expressing the main chemokines involved in the recruitment of immune cells [1–3]. Accumulating evidence indicates that the stromal microenvironment plays an important role in the regulation of the trafficking and phenotype and function regulation of immune cells, including DC [4, 5].

Fibroblasts and other stromal cells from various tissues have been shown to modulate DC differentiation and activation [6–8] and may induce the development/differentiation of DCs from blood monocytes or CD34^+^ hematopoietic precursors [9, 10]. Kitamura et al [11] reported that fibroblasts have a crucial role in regulating both fibrotic and immune responses in the lung by chemokine secretion, favoring dendritic cell trafficking, through TGF-β αvβ8-mediated activation. Dermal fibroblasts were also reported to act as potent immunoregulatory cells able to modulate the maturation of DCs [12–14].

Herein, we aimed to characterize *in vitro* the influence of pulmonary fibroblasts on DC phenotype and function, using co-culture of human pulmonary fibroblasts obtained from patients with idiopathic pulmonary fibrosis (IPF) and from control patients, with immature DCs developed from the precursor cell line MUTZ-3 [15].

### Materials

#### Primary fibroblasts

Primary fibroblasts were derived in Dulbecco’s modified Eagle’s medium (DMEM, Life Technologies-Invitrogen, Courtaboeuf, France) supplemented with 10 % FCS (Life Technologies-Invitrogen) from lung tissue biopsies obtained from 6 patients with IPF (age = 65.4 ± 10.6, 2 F), and from 6 patients with removal of a primary lung tumor without any fibrosis (control patients, age = 53 ± 14, 3 F), according to previous detailed procedures [16]. Fibroblasts were used at passage 5. No significant morphological and phenotypic differences were observed between IPF and control fibroblasts. Informed consent was obtained before lung sampling. The protocol was approved by the ethical committee of Paris-Ile de France 1.

#### MUTZ-3 dendritic cells

The CD34^+^ human MUTZ-3 progenitor cell line (obtained from DSMZ, Braunschweig, Germany) maintained in α-MEM (Life Technologies) supplemented with 20 % FCS and 0.25 ng/ml rhGM-CSF (Gentaur, Bruxelles, Belgium) express CD34 for 80 % and HLA-DR^+^ for 70 %, as previously reported [17]. Generation of dendritic-like cells MUTZ-3 DCs was obtained by culturing MUTZ-3 progenitor (4×10^5^cells/ml) for 7 days in α-MEM with 20 % FCS, 10 ng/ml rhGM-CSF, 10 ng/ml rhIL-4 (Gentaur), and 0.5 ng/ml TNF-α (R&D Systems Europe, Lille, France) (MUTZ − 3 DC medium) [17].

#### Co-cultures

MUTZ-3 DCs (1×10^6^) were co-cultured with fibroblast monolayers (80–90 % confluence) for 48 h in MUTZ-3 DC medium either in 6-well tissue culture plates for direct contact or in Transwell® support. As controls, MUTZ-3 DCs and fibroblasts alone were cultured for 48 h under the same conditions.

#### Mixed Leukocyte Reaction

For mixed leukocyte reaction (MLR), human peripheral blood mononuclear cells were isolated from one leucocyte-enriched buffycoat, obtained from Etablissement Français du Sang (Paris, France), by Lymphocyte Separation Medium^M^ (GE Healthcare, PAA, Austria) density gradient centrifugation, cultured in RPMI 1640 (Gibco, Life Technologies) with 10 % heat inactivated human AB serum (RPMI complete medium) for 2 h in order to eliminate adherent mononuclear cells and resuspended at 1×10^6^ cells/ml RPMI complete medium. MUTZ-3 DCs cultured alone or in the presence of fibroblasts as indicated above were collected, washed once and resuspended in RPMI complete medium also. 2×10^4^ DC were cultured with 1×10^5^ allogeneic T cells in 96-well round-bottom plates, in a final volume of 200 μl for each well. All determinations were performed in sixplicate. Cell proliferation was assessed for 6 days, with during the last 18 h of the culture period, addition of 1 μCi of ^3^H-thymidine (Perkin-Elmer Wallac, Courtabeuf, France) to each well. The cells were then harvested onto FilterMat filters (Wallac) by using the Harvester 96 cell Tomtec harvester, and incorporated radioactivity was determined using a MicroBeta plate reader (Wallac Trilux 1450 MicroBeta Liquid Scintillation Counter & Luminometer, WS-Trilux 1450).

#### Flow Cytometry

Cell surface staining was performed in phosphate-buffered saline (PBS, Life Technologies) (without calcium and magnesium) containing 1 % bovine serum albumin (BSA) (Sigma-Aldrich) using fluorochrome-conjugated monoclonal antibodies recognizing MHC class II and DC-specific molecules: HLA-DR-PE (Pharmingen-BD, L’Isle d’Abeau, France), CD1a-PE, CD34-FITC, CD83-PE, CD86-PE, and CD209-PE (Immunotech-Beckman Coulter, Marseille, France) for 20 min at 4 °C. After two washes in PBS/BSA, the cells were fixed in formaldehyde 1 % and subsequently analyzed using an Epics cytometer (Beckman Coulter). Appropriate gating was used to exclude cell debris. Gating on isotype control staining was used to identify the percentage of cells expressing DC-specific markers. Data were analyzed with FlowJo software (Tree Star, Inc., San Carlos, CA). Analysis of each epitope staining was assessed using both the percentage of positive cells (%) and the mean fluorescence intensity (MFI) of DCs.

#### Cytokine Production

Supernatants from MUTZ-3 DC and fibroblasts either cultured alone or in co-culture were centrifuged (1600 rpm, 10 min, 4 °C) and frozen at −70 °C until assayed using specific enzyme-linked immunosorbent assays (ELISAs) from R&D Systems (Quantikine, R&D Systems, Abingdon, UK).

#### Statistical analysis

Data are presented as means (± SEM), or as individual values and median. Comparisons were performed with Wilcoxon paired nonparametric test for group comparisons or Mann–Whitney U test. *P* values < 0.05 were considered significant. Statistical analysis was performed with Prism 5 (Graphpad Software Inc., La Jolla, CA USA).

## Results

MUTZ-3 DCs obtained after a 7 day-differentiation culture (*n* = 12) were characterized by a low CD34 expression (<15 %), and the expression of CD209 (33.7 ± 1.6 %, mean ± SD) (marker of immaturity), HLA-DR (66.7 ± 2.8 %) and CD86 (59.5 ± 1 %). Few cells, if any, expressed the maturation marker CD83 (0.68 ± 0.23 %). These immature cells can undergo significant maturation by stimulation with 25 ng/ml TNF-α during 48 h as detected by an increased expression of HLA-DR (88.6 ± 1.3 % *p* ≤ 0.001) and the induction of the maturation marker CD83 (47.2 ± 2.1 %).

**Fig. 1:**
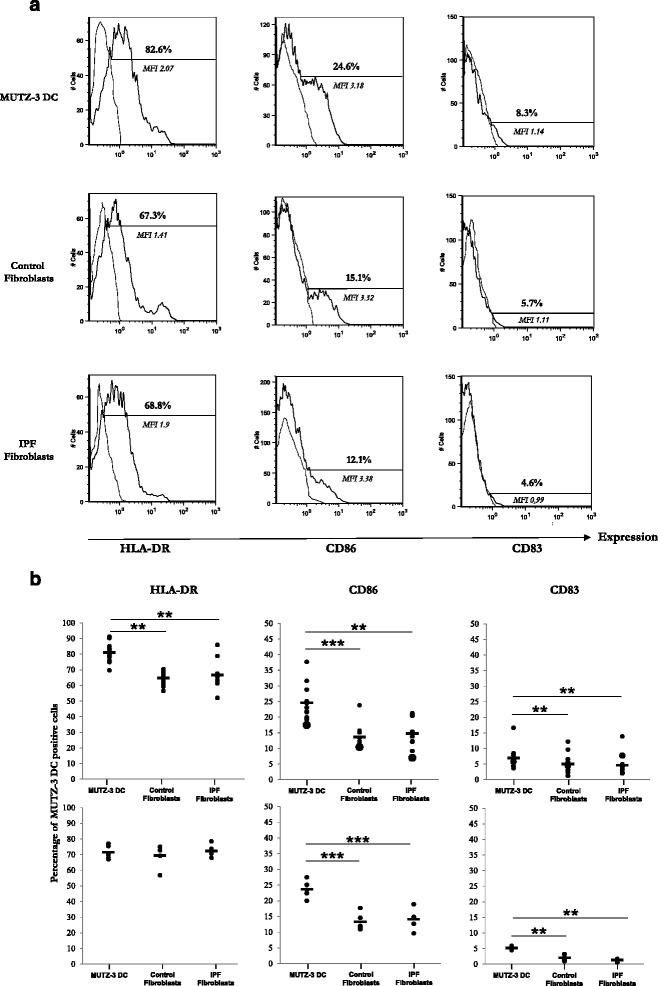
Phenotype of MUTZ-3 DCs alone or cultured with control or IPF fibroblasts for 48 h. Immature MUTZ-3 DCs (1×10^6^) were directly added to fibroblasts monolayers grown to confluence in 6-well tissue culture plates (direct contact) or fibroblasts (low compartment) and MUTZ-3 DCs (high compartment) were separated using modified Boyden chambers (Transwell® permeable support-0.4 μm) to prevent direct cell-cell contact, for 48 h in MUTZ-3 DC medium (Transwell condition). Controls were MUTZ-3 DCs cultured alone for 48 h in the same condition. Flow Cytometry analysis was assessed on DCs stained with monoclonal antibodies HLA-DR-PE (Pharmingen-BD), CD34-FITC, CD83-PE, CD86-PE, and CD209-PE (Immunotech-Beckman Coulter), and acquired with an Epics cytometer (Beckman Coulter). **a** One representative experiment or (**b**) percentage of positive cells for HLA-DR, CD86, and CD83 expression, represented as mean (dark horizontal bars) and individual values (*n* = 10) for direct contact co-cultures (upper panels) and Transwell® co-cultures (lower panels). MFI: Mean fluorescence intensity. **p < 0.01, ***p < 0.001. Statistical comparisons were performed with Wilcoxon paired nonparametric test for group comparisons or Kruskal-Wallis for unpaired nonparametric test, using Prism 5 (Graphpad Software Inc.)

Co-culture of lung fibroblasts with immature MUTZ3-DCs for 48 h induced a 20 % decrease of HLA-DR expression and a 40 % decrease in CD83 and CD86 expression by DCs (Fig. 1). CD209 expression by DC was not significantly altered by co-culture (data not shown). It is noteworthy that IPF and control fibroblasts had a similar inhibitory effect on DCs.

In accordance with the decreased expression of activation/maturation markers, co-culture of lung fibroblasts from either control or IPF patients and MUTZ-3 DCs induced a significant 30 % reduction of lymphocyte allostimulatory activity as compared to that observed for MUTZ-3 DCs cultured alone (Table 1).

**Table 1:** Mixed leukocyte reaction (MLR)

Conditions	MUTZ-3 DC medium	Contact Co-culture	
	medium	Control Fibro	IPF Fibro
Lymphocyte Proliferation (mean cpm ± SD, *n* = 3)	56,870 ± 6110	40,236 ± 6047	34,322 ± 2843
*p* value (versus MUTZ-3 DC)		0.02	0.01
Conditions	MUTZ-3 DC medium	Transwell Co-culture	
	medium	Control Fibro	IPF Fibro
Lymphocyte Proliferation (mean cpm ± SD, *n* = 3)	36,967 ± 4942	29,328 ± 3215	26,890 ± 6564
*p* value (versus MUTZ-3 DC)		0.04	0.05

MLR was performed by culturing with 100 000 lymphocytes (depleted of adherent mononuclear cells by a 2 h-lasting culture) and 20 000 MUTZ-3 DCs previously cultured alone (medium) or co-cultured with control or IPF fibroblasts for 48 h (as in Fig. 1) in 200 μl RPMI complete medium (*n* = 6 for each condition) for 6 days During the last 18 h, 1 μCi of 3H-thymidine (Perkin-Elmer Wallac) was added per well. Incorporated radioactivity was determined using a MicroBeta plate reader (Wallac WS-Trilux 1450

Results are presented as mean (±SD) lymphocyte proliferation induced by MUTZ-3 DCs previously alone (medium) or co-cultured with control or IPF fibroblasts either in direct contact or in transwell conditions during 48 h before MLR

3H-thymidine incorporation of 105 lymphocytes alone was 605 ± 436 cpm and 3H-thymidine incorporation of 20 000 MUTZ3 DC alone was 138 ± 59 cpm, (mean ± SD, *n* = 6)

Lung fibroblasts might alter MUTZ-3 DC phenotype by either direct cell-cell contact or soluble factors. Of interest, MUTZ-3 DCs co-cultured with fibroblasts using modified Boyden chambers (Transwell® support) to prevent direct cell-cell contact did also show significant decrease in DC CD86 and CD83 markers (Fig. 1b*lower panels*), decreases that were not significantly different from those observed in cell-cell contact conditions (upper panels).

**Fig. 2:**
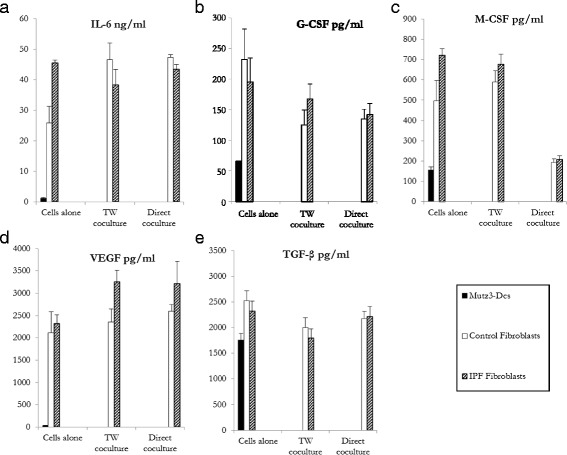
Production of the cytokines IL-6, G-CSF, M-CSF, VEGF and TGF-β by MUTZ-3 DC (dark), control fibroblasts (white) or IPF (dashed) fibroblasts, cultured either alone or in direct contact or using Transwell (TW) support. Mean levels (± SEM, *n* = 6) of (**a**) IL-6, (**b**) G-CSF, (**c**) M-CSF, (**d**) VEGF, and (**e**) TGF-β, assessed by ELISA after 48 h of both culture types. Cytokine levels were analysed using the normality test. The Mann-Whitney *U* and Student *t* test were used to compare the mean values of cytokines between patients and control subjects. TW: transwell. *p < 0.05

Numerous cytokines such as IL-6, G-CSF, M-CSF, VEGF, and TGF-β [18, 19] are produced by fibroblasts in the microenvironment and may inhibit MUTZ-3 DC maturation and function. Accordingly, we detected measurable levels of these cytokines in the culture supernatants of control and IPF fibroblasts, either alone or in co-culture (Fig. 2). Although IL6 production was lower in control fibroblasts alone compared to IPF ones, no differences were observed between the two fibroblast types when co-cultured with MUTZ3-DC, either in contact or not. Noteworthy, a significant decrease in MCSF production was detected for both populations when cultured in contact with MUTZ-3 cells.

## Discussion

The present study demonstrates that lung fibroblasts co-cultured with DCs, differentiated from CD34^+^ MUTZ-3 precursors and closely resembling monocyte-derived DCs in terms of morphology, phenotype and function [15, 17, 20–22], induced a reduced MHC classII, CD86, and CD83 expression by DCs, with a decrease in their T-cell stimulatory activity. These findings support the notion that lung fibroblasts are effective in maintaining DCs in an immature state within fibrotic lesions. Our results are in line with recent data showing that human dermal fibroblasts are potent immunoregulatory cells inhibiting allogeneic T-cell activation by DCs [11]. Moreover, dermal fibroblasts inhibited T-cell proliferation in response to DCs with similar potency to bone marrow-derived mesenchymal stem cells (MSC), suggesting that immunosuppression is a general property of stromal cells [11, 23].

The absence of difference between IPF and control fibroblasts in our study suggests that the inhibitory effect of lung fibroblasts on DC differentiation and function is an intrinsic property of this cell type, independently of the physiological context. This possibility, which fits well with the recently documented immunoregulatory function of MSC [24], needs to be further assessed with fibroblasts isolated from various other tissues.

## Conclusion

In conclusion, our findings suggest that lung fibroblasts by their ability to modulate DC differentiation and function, are able to participate in pulmonary inflammatory and immune responses. Whereas their role in production of extracellular matrix proteins is a key factor in healing and remodeling, their likely interaction with lung DCs might be also considered as critical in inflammatory lung disorders.

## Abbreviations

DC: Dendritic cells
IPF: Idiopathic pulmonary fibrosis
MSC: Mesenchymal stem cells
